# Instrumented Mouthguards in Elite-Level Men’s and Women’s Rugby Union: The Incidence and Propensity of Head Acceleration Events in Matches

**DOI:** 10.1007/s40279-023-01953-7

**Published:** 2023-10-31

**Authors:** James Tooby, James Woodward, Ross Tucker, Ben Jones, Éanna Falvey, Danielle Salmon, Melanie Dawn Bussey, Lindsay Starling, Gregory Tierney

**Affiliations:** 1 Carnegie Applied Rugby Research (CARR) Centre, Carnegie School of Sport, Leeds Beckett University, Leeds, UK; 2 Sport and Exercise Sciences Research Institute, Ulster University, Belfast, UK; 3 Department of Sport Science, Institute of Sport and Exercise Medicine, University of Stellenbosch, Stellenbosch, South Africa; 4 World Rugby, 8-10 Pembroke St., Dublin, Ireland; 5 Division of Physiological Sciences and Health Through Physical Activity, Department of Human Biology, Faculty of Health Sciences, Lifestyle and Sport Research Centre, University of Cape Town, Cape Town, South Africa; 6 England Performance Unit, Rugby Football League, Manchester, UK; 7 Premiership Rugby, London, UK; 8 Faculty of Health Sciences, School of Behavioural and Health Sciences, Australian Catholic University, Brisbane, QLD Australia; 9 School of Medicine & Health, University College Cork, Cork, Ireland; 10 New Zealand Rugby, Auckland, New Zealand; 11 School of Physical Education Sport and Exercise Sciences, University of Otago, Dunedin, New Zealand

## Abstract

**Objectives:**

The aim of this study was to examine head acceleration event (HAE) propensity and incidence during elite-level men’s and women’s rugby union matches.

**Methods:**

Instrumented mouthguards (iMGs) were fitted in 92 male and 72 female players from nine elite-level clubs and three international teams. Data were collected during 406 player matches (239 male, 167 female) using iMGs and video analysis. Incidence was calculated as the number of HAEs per player hour and propensity as the proportion of contact events resulting in an HAE at a range of linear and angular thresholds.

**Results:**

HAE incidence above 10 g was 22.7 and 13.2 per hour in men’s forwards and backs and 11.8 and 7.2 per hour in women’s forwards and backs, respectively. Propensity varied by contact event, with 35.6% and 35.4% of men’s tackles and carries and 23.1% and 19.6% of women’s tackles and carries producing HAEs above 1.0 krad/s^2^. Tackles produced significantly more HAEs than carries, and incidence was greater in forwards compared with backs for both sexes and in men compared with women. Women's forwards were 1.6 times more likely to experience a medium-magnitude HAE from a carry than women's backs. Propensity was similar from tackles and carries, and between positional groups, while significantly higher in men than women. The initial collision stage of the tackle had a higher propensity than other stages.

**Conclusion:**

This study quantifies HAE exposures in elite rugby union players using iMGs. Most contact events in rugby union resulted in lower-magnitude HAEs, while higher-magnitude HAEs were comparatively rare. An HAE above 40 g occurred once every 60–100 min in men and 200–300 min in women. Future research on mechanisms for HAEs may inform strategies aimed at reducing HAEs.

**Supplementary Information:**

The online version contains supplementary material available at 10.1007/s40279-023-01953-7.

## Key Points

**Table:** 

Head acceleration event incidence describes the exposure of elite rugby union players for the first time.
The relative risk of contact events to result in head acceleration events provided by this study has potential implications for strategies aimed at reducing head acceleration event exposure in rugby.
The tackle should remain a focus of head acceleration mitigation strategies with consideration for both the tackler and the ball carrier, but attention may also be required for rucks and other contact events.

## Introduction

Rugby union is a contact sport involving collisions from tackles, carries, rucks and other contact events (e.g., mauls and scrums) [1]. Consequently, rugby union players are at risk of sustaining injuries, with concussion being the most prevalent injury in the elite game [2].

Research has identified risk factors for head injuries during the tackle [3–5] and law modifications to reduce head injuries have been trialled [6, 7]. These previous studies have examined head impacts sufficient to cause concussion or require the removal of a player for a head injury assessment (HIA) [3]. During tackles that do not result in an injury, players may also experience a head acceleration event (HAE), which is a short-duration head acceleration response to an external collision force, caused by either direct contact to the head or indirectly through force applied to the body [4, 8–10]. Acute and cumulative HAEs [6] have been suggested to have negative effects on cognition and other physiological outcomes [11–14], and so understanding HAE incidence and propensity is crucial to provide insight into players' overall exposure to HAEs, and may also guide the search for effective prevention initiatives.

Historically, sensors measuring HAEs have been compromised by soft-tissue artefacts, where the sensor moves independently of the player’s head (e.g., skull-cap and skin-based sensors) [15]. The advent of instrumented mouthguards (iMGs) enables valid and reliable measurement of linear and rotational head kinematics [16–18] because iMGs have demonstrated superior coupling to the head [15]. Implementing iMGs in rugby offers a unique opportunity to understand HAEs during match play. The inclusion of male and female data is also essential to further explore differences in HAE mechanisms so that sex-specific mitigation strategies can be explored [19].

The aim of this study was to utilise iMGs and video analysis to describe the occurrence of HAEs during elite-level men’s and women’s rugby matches. Specifically, the incidence of HAEs per player hour and the propensity of tackles, carries and rucks to result in linear and rotational HAEs are presented across a range of magnitudes. Additionally, we conducted comparisons between playing positions and by sex. The objective was to provide benchmarks for HAE exposure in rugby union, while identifying potential areas for developing effective mitigation strategies for reducing the frequency and magnitude of HAEs in rugby.

## Methods

### Study Design and Participants

A prospective observational cohort study was conducted in players from nine elite rugby clubs (92 male and 40 female) and three international teams (32 female players) during the 2021/22 season across multiple elite-rugby competitions (Farah Palmer Cup, New Zealand; The National Provincial Championship, New Zealand; The Premiership, United Kingdom; United Rugby Championship; Italy, Scotland, Wales, Ireland, South Africa; Top 14, France). Participation was voluntary and all players from each club were offered iMGs. Ethics approval was received from the University of Ulster's Research Ethics Committee (UREC; #REC-21–0061) and the University of Otago Human Ethics Committee (REF: H21-056). Custom-fit iMG devices were provided to ten teams (92 male, 42 female) through 3D dental scans, while two teams (30 female) received boil-and-bite iMG fitted by a dental practitioner. All iMGs were supplied by Prevent Biometrics (Minneapolis, MN, USA). The iMGs contain an accelerometer and gyroscope that sample at 3200 Hz with measured ranges of ± 200 g and ± 35 rad/s. Infrared proximity sensors assessed coupling of the mouthguard to the upper dentition. Previous studies have shown the laboratory and on-field validity of the Prevent Biometrics iMG, with concordance correlation coefficient values of 0.98 [16] and 0.89 [17] for the accuracy of kinematics of custom-fit and boil-and-bite iMGs in laboratory-based validations, respectively. Positive predictive values (PPV) and sensitivity values of 0.94 and 0.75 during on-field video-verification validation of the custom-fit iMGs were also found [16].

To further assess the sensitivity of the iMGs, a false-negative validation was conducted. A video analyst blinded to iMG data identified 258 head impacts on match video footage, of which 223 had measured HAEs, resulting in a sensitivity of 0.86. The trigger mechanism for iMGs was set to capture HAEs when a single sample on the accelerometer exceeded 8 g on any axis, recording 10 ms of pre- and 40 ms of post-trigger data. Peak linear acceleration (PLA), peak angular acceleration (PAA) and peak change in angular velocity (ΔPAV) were extracted from each HAE. Linear head kinematics were transformed from the iMG location to the head centre of gravity (CG). An in-house Prevent Biometrics algorithm classified the level of noise/artefact in the kinematic signal as minimal (class 0, *n* = 9597), moderate (class 1, *n* = 695), or severe (class 2, *n* = 322). A 4-pole, zero-phase, low-pass Butterworth filter was applied to each signal with cut-off frequencies (− 6 dB) of 200, 100 and 50 Hz for class 0, 1 and 2 HAEs, respectively.

Data were collected from 406 player matches (239 male, 167 female player matches) across 43 matches using iMGs and video analysis. Qualitative video analysis of synchronised HAEs identified the contact event (tackle, carry, ruck) associated with each HAE and contact stage associated with each tackle and carry HAE (initial collision, secondary contact, grounding, breakdown) [20]. Opta match event data provided by StatsPerform (Chicago, IL, USA) also included tackles, carries and rucks. A tackle was defined as an attempt to halt the progress of an opponent, a carry as an engagement of an opponent whilst carrying the ball, and a ruck contact event as a player entering a breakdown after it had been set. Other contact events were identified using consensus definitions [1].

Opta contact events were linked to HAEs if they occurred within 10 s of the contact event timestamp for the known player and if the contact event matched based on qualitative video analysis of each HAE. Proximity sensors provided timestamps of on-the-teeth periods that were time-synchronised to video timestamps of contact events from Opta data to confirm that the iMG was being worn during each analysed contact event. Only contact events that corresponded with an on-the-teeth period for the instrumented player were used in propensity calculations (*n* = 7264, 74.5%) and only player matches where the instrumented player wore their iMG for at least 90% of their contact events were used (*n* = 265, 65.3%). A total of 95 player matches were removed due to missing proximity sensor logs. After removing ineligible players and player matches, the cohort consisted of 65 men’s players (42 forwards, 23 backs) and 64 women’s players (36 forwards, 28 backs).

### Recording Threshold

The trigger threshold, defined as the linear acceleration at which an HAE is captured, has previously been found to capture low-magnitude HAEs caused by non-contact events such as running (i.e., false-positives) [16]. Consequently, a recording threshold (threshold above which HAE are reported) can be applied to minimise false-positive capture. Previously, a recording threshold of 10 g has been used [21]. However, HAEs from contact events have been reported below 10 g in rugby league [16, 20]. Therefore, we sought to apply a lower recording threshold to appropriately capture HAEs from contact events only. This optimal threshold was determined by identifying false-positives in a validity analysis on a subset of HAEs (*n* = 6055). False-positive performance was measured using PPVs with 95% confidence intervals (CIs) [16]. Baseline PPV (i.e., no recording threshold) was 0.94 (95% CI 0.91–0.96). Applying a combined recording threshold of 5 g and 0.4 krad/s^2^ improved PPV to 0.99 (95% CI 0.97–1.00) while removing 8.4% (95% CI 7.2–9.6) of contact event HAEs. These improvements were due to 94.5% (*n* = 293) of non-contact events resulting in a PAA below 0.4 krad/s^2^ and 44.4% (*n* = 20) of non-contact events resulting in a PLA lower than 5 g. Consequently, we applied a combined recording threshold to remove all HAEs with magnitudes below 5 g or 0.4 krad/s^2^ from subsequent analysis (*n* = 1520). A trigger threshold of 8 g can record HAEs below 8 g because the trigger threshold is applied to kinematics recorded at the iMG location, however the magnitude of an HAE is reported after these kinematics have been transformed to the head CG.

### Statistical Analysis

Incidence was calculated as the number of HAEs per match player hour. Playing time was obtained from Opta data for each player match and did not include time in which the clock was stopped (i.e., if a player played the entire match, their playing time was 80 min). Propensity values were calculated by dividing the number of events that resulted in an HAE at each threshold by the total number of events the player was involved in while wearing an iMG for each phase of play. Mean values were calculated across players and 95% CIs were estimated using a bootstrapping procedure, as sample sizes varied from 26 to 47. The dataset was randomly resampled 2500 times and the 2.5th and 97.5th percentile of resampled means were used as the lower and upper bounds of CI, respectively.

Mean incidence and propensity, along with 95% CIs, were calculated across a range of magnitude thresholds. A single contact may result in multiple HAEs due to multiple collisions; in these cases, propensity was calculated using the HAE with the greatest magnitude. For statistical comparisons of HAEs, incidence and propensity were collected between three arbitrary magnitude bands based on PLA and PAA thresholds: lower magnitude (PLA < 10 g and PAA < 1.0 krad/s^2^), medium magnitude (PLA between 10 and 30 g*,* and PAA between 1.0 krad/s^2^ and 2.0 krad/s^2^) and higher magnitude (PLA ≥ 30 g or PAA ≥ 2.0 krad/s^2^). If no HAE occurred during a contact event it was assumed to fall within the lower-magnitude band, due to proximity sensors indicating that the iMG was being worn during the event. Ratios between incidences and propensities were calculated to compare between events or groups, and a significant difference was assumed if the CIs did not overlap.

## Results

**Fig. 1:**
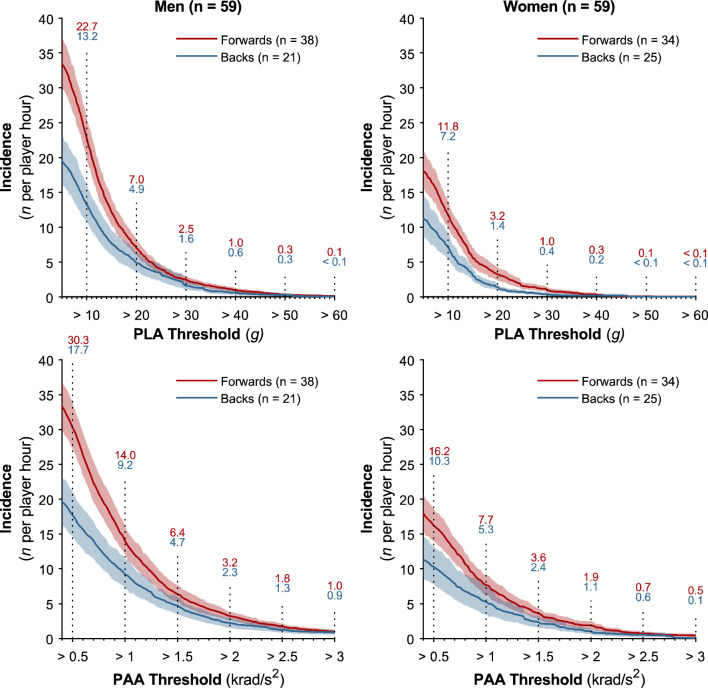
The incidence of HAEs for men’s and women’s forwards and backs across PLA (5–60 g) and PAA thresholds (0.4–3.0 krad/s^2^). *Shaded regions* indicate 95% CI. The number of players available to calculate each curve is shown as *n*. Text labels are added at intervals along the curve. Supplementary Fig. 1 in the electronic supplementary material (ESM) illustrates findings using ΔPAV thresholds. *CI* confidence interval, *HAEs* head acceleration events, *PAA* peak angular acceleration, *PLA* peak linear acceleration, *ΔPAV* peak change in angular velocity

**Fig. 2:**
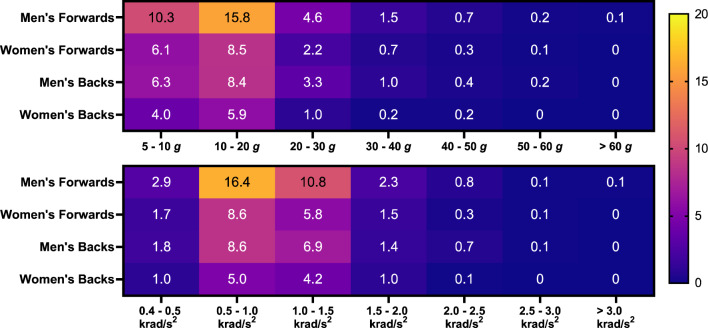
Incidence values between PLA and PAA thresholds for men’s and women’s forwards and backs. Supplementary Fig. 2 in the ESM illustrates findings using ΔPAV thresholds. *PAA* peak angular acceleration, *PLA* peak linear acceleration, *ΔPAV* peak change in angular velocity

Incidence values were computed from a total of 14,898 match minutes (52.0% men’s, 48.0% women’s) across 265 player matches (53.2% men’s, 46.8% women’s). The incidence of PLA and PAA HAEs above a range of thresholds in men and women is shown in Fig. 1, while Fig. 2 displays the PLA and PAA incidence between these thresholds. The incidence of HAEs above 10 g was 22.7 and 13.2 HAEs per player hour for men’s forwards and backs, respectively, while PAA incidence above 1.0 krad/s^2^ was 14.0 and 9.2 HAEs per player hour for men’s forwards and backs, respectively (Fig. 1). Incidence of HAEs above 40 g was 1.0 and 0.6 per player hour and 3.2 and 2.3 for HAEs above 2.0 krad/s^2^ for forwards and backs, respectively.

In women’s forwards and backs, the PLA incidence above 10 g was 11.8 and 7.2 per player hour, respectively, while the PLA incidence above 40 g was 0.3 and 0.2 HAEs per player hour. For HAEs above 1.0 krad/s^2^, the incidence was 7.7 and 5.3 HAEs per player hour for women’s forwards and backs, while for HAEs above 2.0 krad/s^2^, the incidence was 1.9 and 1.1 HAEs per player hour, respectively. Incidence decreased as HAE magnitude increased (Fig. 2). The incidence of HAEs below 10 g and 0.5 krad/s^2^ was lower than HAEs between 10 and 20 g and 0.5 and 1.0 krad/s^2^ due to low-magnitude HAEs failing to exceed the trigger threshold and therefore not being recorded by iMGs.

Video analysis of instrumented players identified 3830 and 3461 iMG-measured contact events in men and women players. Table 1 shows the mean number of tackles, carries and rucks completed per player hour, the total number of measured events and the proportion of measured events resulting in a given number of HAEs for each player group. When combining all men’s contact events, 51.1% resulted in no recorded HAE (i.e., did not exceed the trigger threshold), 28.6% resulted in a single HAE and 20.3% resulted in multiple HAEs. In women, 67.8% of all contact events resulted in no HAEs, 23.0% resulted in a single event and 9.2% resulted in multiple HAEs.

**Table 1:** The mean number of each contact event type completed per player hour, the total number of measured events and the total number of each contact event type to result in a given number of HAEs for men’s and women’s forwards and backs. Events were measured if proximity sensor readings indicated the iMG was worn during the event

Contact event	Mean *n* of contact events completed (per player hour)	Total *n* of measured contact events	*n* (%) of HAEs per measured event			
			0	1	2	3 +
Men's forwards						
Tackles	10.4 (9.5–11.2)	849	328 (38.6)	296 (34.9)	137 (16.1)	88 (10.4)
Carries	5.4 (4.7–6.0)	437	148 (33.9)	158 (36.2)	75 (17.2)	56 (12.8)
Rucks	18.8 (16.4–21.3)	1436	924 (64.3)	350 (24.4)	123 (8.6)	39 (2.7)
Women's forwards						
Tackles	11.0 (9.6–12.6)	847	485 (57.3)	235 (27.7)	98 (11.6)	29 (3.4)
Carries	5.3 (4.3–6.0)	389	211 (54.2)	117 (30.1)	48 (12.3)	13 (3.3)
Rucks	18.2 (15.6–20.8)	1225	981 (80.1)	194 (15.8)	44 (3.6)	6 (0.5)
Men's backs						
Tackles	7.3 (6.4–8.3)	398	162 (40.7)	109 (27.4)	77 (19.4)	50 (12.6)
Carries	5.1 (4.4–5.9)	312	144 (46.1)	87 (27.9)	45 (14.4)	36 (11.5)
Rucks	5.9 (4.9–6.9)	371	237 (63.9)	86 (23.2)	32 (8.6)	16 (4.3)
Women's backs						
Tackles	6.6 (5.6–7.7)	399	230 (57.6)	124 (31.1)	36 (9.0)	9 (2.3)
Carries	5.3 (4.6–5.9)	291	194 (66.7)	72 (24.7)	19 (6.5)	6 (2.1)
Rucks	6.2 (4.9–7.3)	310	246 (79.3)	53 (17.1)	10 (3.2)	1 (0.3)

**Fig. 3:**
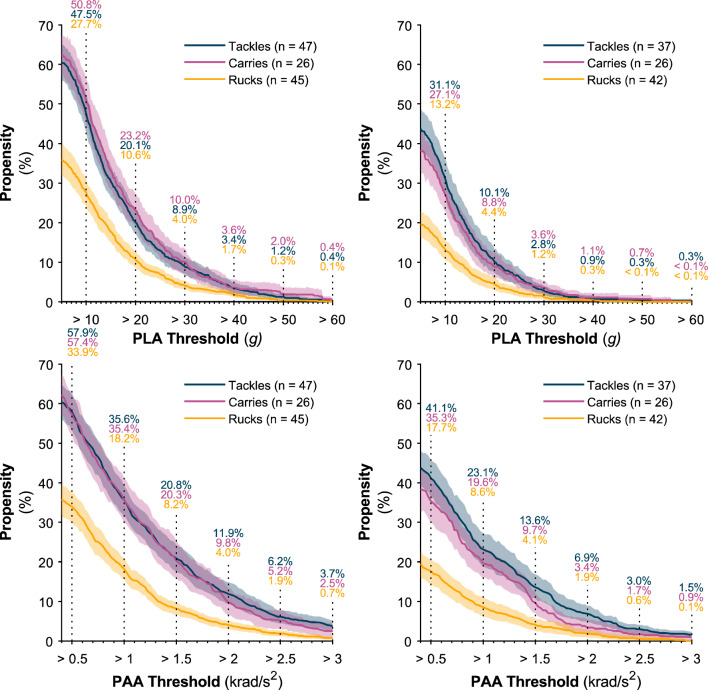
The propensity of tackles, carries and rucks to result in at least one HAE exceeding a given PLA (5–60 g) or PAA threshold (0.4–3.0 krad/s^2^) for men’s and women’s players. *Shaded regions* indicate 95% CI. The number of players available to calculate each curve is shown as *n*. Text labels are added at intervals along the curve. Supplementary Fig. 1 in the ESM illustrates findings using a ΔPAV threshold. *CI* confidence interval, *HAEs* head acceleration events, *PAA* peak angular acceleration, *PLA* peak linear acceleration, *ΔPAV* peak change in angular velocity

**Fig. 4:**
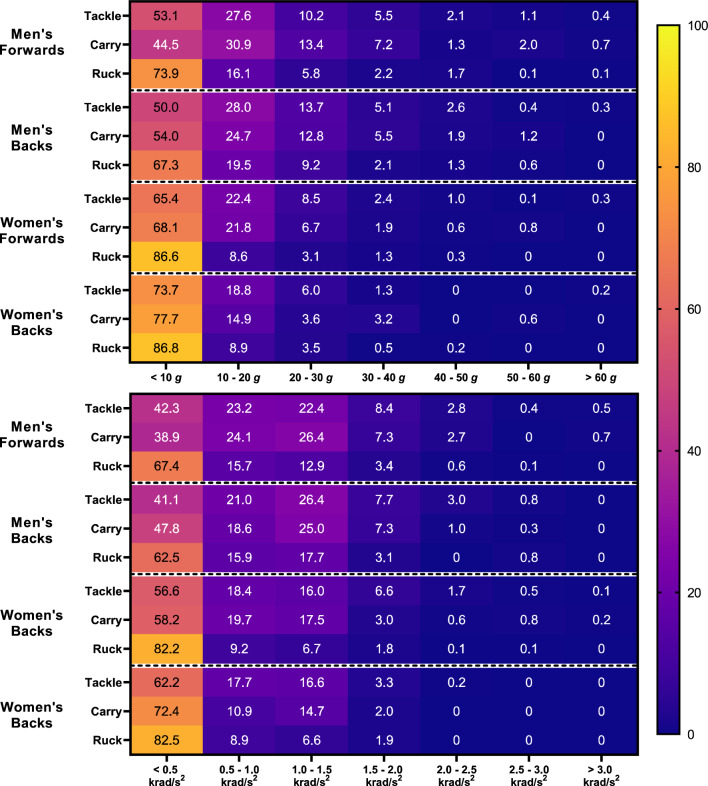
Propensity values between PLA and PAA thresholds for men’s and women’s forwards and backs. Supplementary Fig. 3 illustrates findings using a ΔPAV threshold (see ESM). *PAA* peak angular acceleration, *PLA* peak linear acceleration, *ΔPAV* peak change in angular velocity

Propensity values are presented above thresholds in Fig. 3 and between thresholds in Fig. 4. In men, 50.8% of carries resulted in an HAE above 10 g and 35.4% exceeded 1.0 krad/s^2^*,* while 3.6% resulted in HAEs above 40 g and 9.8% exceeded 2.0 krad/s^2^. In women, 27.1% of carries produced an HAE above 10 g and 19.6% exceeded 1.0 krad/s^2^, while 1.1% resulted in an HAE above 40 g and 3.4% above 2.0 krad/s^2^. Both PLA and PAA propensity decreased as magnitude increased.

**Fig. 5:**
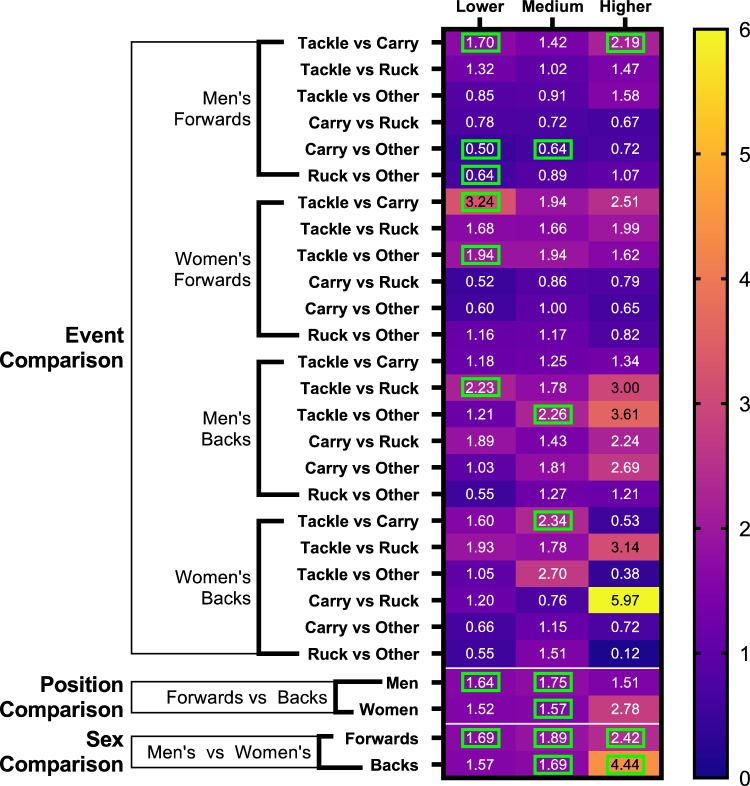
Incidence ratios of HAEs caused by tackles, carries, rucks and other contact events within lower-, medium- and higher-magnitude bands. Comparisons between events, positions and sexes are included. Significant comparisons are indicated by *green boxes*. *HAEs* head acceleration events

**Fig. 6:**
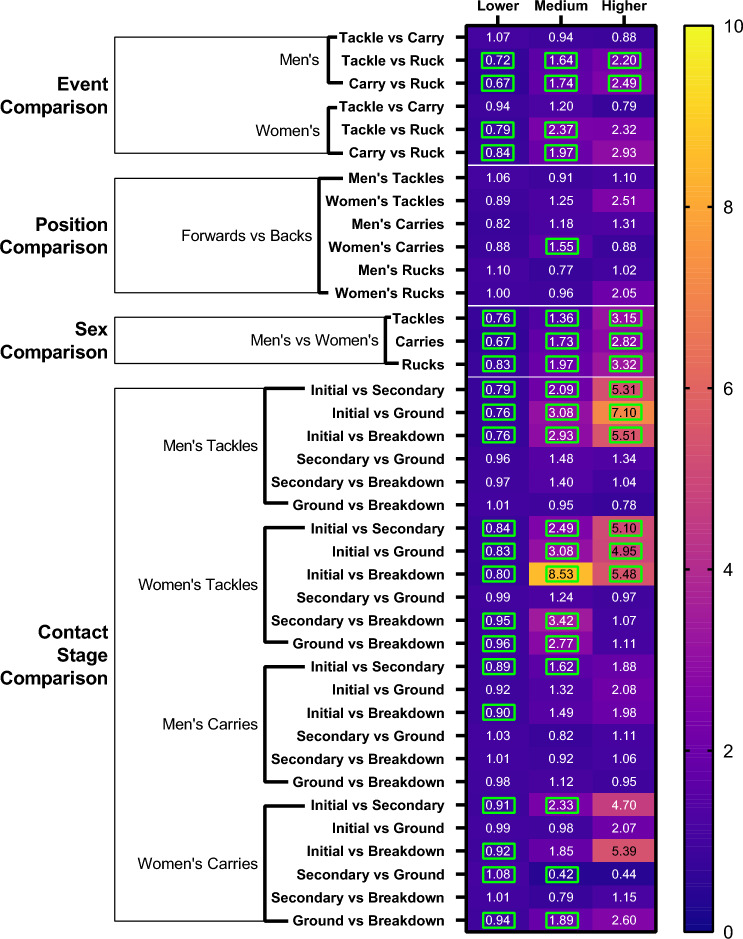
Propensity ratios of tackles, carries and rucks to result in a maximum-magnitude HAE within lower-, medium- and higher-magnitude bands. If no HAE was recorded during a contact event then the maximum HAE was considered to be within the lower band. Comparisons between events, positions and sexes, and contact stages are included. Significant comparisons are indicated by *green boxes*. *HAEs* head acceleration events

Statistical comparisons were conducted to compare incidence and propensity of contact event types by playing position and by sex. For this analysis, HAEs were categorised as lower, medium and higher magnitude. Figures 5 and 6 present the ratios between groups of interest for incidence and propensity, respectively.

Tackles produced significantly more HAEs than carries in all player groups except men’s backs. For example, in men’s forwards, higher-magnitude HAEs occurred 2.19 times more frequently from tackles than from carries (Fig. 5). Tackles and rucks produced a similar HAE incidence in men’s and women’s forwards and in women’s backs, whereas in men’s backs tackles were 2.23 times more likely to cause lower-magnitude HAEs than rucks.

Playing position also influenced HAE incidence. Men’s forwards experienced 1.6 and 1.8 times the rate of lower- and medium-magnitude HAEs than backs, while women’s forwards experienced medium-magnitude HAEs 1.6 times more often than backs (Fig. 5). Incidence was significantly higher in men than in women, with forwards 1.7, 1.9 and 2.4 times more likely to experience lower-, medium- and higher-magnitude HAEs than women forwards. In backs, medium- and higher-magnitude HAE incidence was significantly greater in men than in women.

The propensity of tackles to result in HAEs was similar to carries in both men’s and women’s players (Fig. 6). Rucks were significantly more likely to result in lower-magnitude HAEs than tackles and carries (if no HAE occurred during a contact event during propensity comparisons it was assumed to fall within the lower-magnitude band), while tackles and carries were significantly more likely to result in medium- and higher-magnitude HAEs than rucks.

There were no significant differences in HAE propensity between forwards and backs for any contact type, with the exception of women’s forwards, who were 1.6 times more likely to experience a medium-magnitude HAE from a carry than women’s backs. Following all contact event types, men’s players were significantly less likely to experience a lower-magnitude HAE and significantly more likely to experience medium- and higher-magnitude HAEs than women’s players (Fig. 6).

During tackles and carries there are various stages in which HAEs can occur. The initial collision was significantly more likely to result in medium- and higher-magnitude HAEs than all other stages in both men’s and women’s tackles. For example, men’s players were 5.3–7.1 times more likely to experience a higher-magnitude HAE from the initial collision than from other stages. In women’s tackles, the breakdown was significantly less likely to result in medium- and higher-magnitude HAEs than secondary contact and grounding stages, while in men’s tackles, there were no significant differences between the propensity of secondary contact, grounding and breakdown stages. Unlike during tackles, the initial collision in a carry did not have a significantly higher propensity than other stages to result in HAEs, with the exception of the secondary contact stage, which was significantly less likely to result in an HAE in both men and women. In women’s carries, the grounding stage was significantly more likely to result in medium-magnitude HAEs than both the secondary and breakdown stages, and had a similar propensity to the initial collision.

## Discussion

The aim of this study was to utilise iMGs and video analysis to describe the incidence and propensity of HAEs during elite-level men’s and women’s rugby matches. Based on 14,898 match minutes and 7264 iMG-measured contact events, the majority of contact events did not register HAEs above the thresholds used in the present study (Table 1), and the incidence of HAEs decreased significantly as HAE magnitude increased, for both linear and angular acceleration. Higher magnitude HAEs were comparatively rare, with an HAE exceeding 40 g occurring every 30 tackles, 28 carries and 59 rucks in men, and every 111 tackles, 91 carries and 333 rucks in women. In total, an HAE > 40 g occurred every 60 and 100 min for men’s forwards and backs, respectively, and every 200 and 300 min for women’s forwards and backs.

Additionally, statistical analysis was used to compare incidence and propensity between groups of interest. Key findings from this analysis included a higher, though not always significant, HAE incidence from tackles than carries, forwards experiencing HAEs at a greater rate than backs and men’s players experiencing HAEs at a greater rate than women’s players. With respect to propensity, tackles and carries were equally likely to result in HAEs, the propensity of contact events to result in HAEs was similar between positional groups, men’s contact events had a higher propensity than women’s contact events and the initial collision stage of the tackle had a higher propensity than other stages.

Incidence, measured as HAEs per player hour, can be considered a product of how many contact events a player is exposed to, and the propensity of each of those contact events to result in HAEs. This product accounts for the present findings. For example, men experienced more HAEs per match hour than women, despite having a similar overall rate of contact events to women (Table 1). Therefore, the greater incidence in men can be explained by the higher propensity of each contact event to result in an HAE in men than in women. The mechanism for this difference in propensity requires future mechanistic studies, but may be the result of physical and/or technical differences between men’s and women’s contact events.

Conversely, HAE incidence was significantly greater in forwards than in backs despite no significant difference in propensity, meaning that the incidence difference is created by forwards completing a higher number of contact events per hour than backs (Table 1), rather than inherent differences between forwards and backs in contact.

Ultimately, the reduction of HAE exposure will be measured by lowering the incidence, however this can be achieved either by reducing exposure to the risk events, or by modifying the risk events such that their propensity to cause an HAE is reduced. By examining both incidence and propensity, we are better able to understand and identify how HAE risk may be reduced. Propensity may be reduced by identifying which behaviours increase the risk of HAEs, and then modifying player’s technique, conditioning levels and possibly laws to avoid these higher-risk situations. Exposure to the risk event can be reduced by limiting match exposure, or reducing the number of contact events in a given period. Both areas are important for future research to ascertain which can be most effective in reducing HAE exposure.

Given the assumption that HAEs should be mitigated where possible, more targeted interventions can be explored in rugby union. Mitigation strategies may aim to reduce HAEs from the most frequently occurring contact events, or the contact events with the highest risk of high-magnitude HAEs. In both cases, tackles should continue to be the focus of interventions [6, 7]. Specifically, the initial collision stage of the tackle was significantly more likely to result in HAEs than other stages. These findings may also inform position- and sex-specific match exposure guidelines based on HAE exposure, which at present are based on overall injury risk models [22].

Finally, these data provide intriguing contrasts compared with previous studies of HIA and concussion risk. In the present study, tackles and carries exhibited a similar propensity to result in HAEs at all magnitudes (Figs. 3 and 4). However, previous research has found that in men, tackles had a higher propensity to result in HIAs and concussions than carries [4, 5, 23]. In a smaller sample (*n* = 69) of women’s concussions, the ball carrier experienced slightly more concussions than the tackler (ratio 1.3 ball carrier to tackler) [24]. This raises the possibility that future mitigation strategies designed to reduce HIA and concussion incidence may not necessarily reduce HAE incidence, and vice versa. Therefore, monitoring the incidence and propensity of both HIAs and HAEs following the introduction of head injury mitigation policies is essential to identify potential unintended consequences that may occur to one outcome when attempting to mitigate the other (i.e., HIAs vs HAEs). Moreover, more research is needed to investigate the clinical outcomes of HAEs across different magnitudes to inform which HAEs need to be mitigated.

## Limitations

This study was limited to examining HAEs during match play and did not consider HAEs that occur during training sessions. It is important to assess HAE exposure during both match play and training to gain a comprehensive understanding of the cumulative HAE risk in rugby union. Secondly, it is essential to acknowledge the limitations of iMGs. The filtering of kinematics was conducted as part of Prevent Biometrics’ in-house processes and has therefore been incorporated into previous validations of the entire iMG system [16–18]. However, these kinematic filters, as well as the proximity sensors, lack individual validation. Additionally, our study revealed a false-negative rate of 14%, indicating that the reported exposures are likely to be underestimated. Prior simulations [27] have suggested that false negatives may be influenced by a bias introduced through linear acceleration trigger mechanisms. Thirdly, the study did not address the influence of tackle, carry or ruck technique on HAE incidence and propensity. This has been recognised as a significant risk factor for injury, and further studies should aim to assess the effect of technique on HAE risk to inform coaching and rule changes. Finally, this study utilises peak resultant head kinematics (PLA, PAA and ΔPAV) which do not consider directionality and temporal data (e.g., pulse duration) from the kinematic signals which are likely to be critical to injury risk [25]. Finite element brain model-based metrics may improve HAE studies in the future, however, differences in model predictions exist [26].

## Conclusion

This novel study utilised iMG technology and video analysis to quantify HAEs in elite-level men’s and women’s rugby union. Typical HAE exposures of elite rugby union players are reported and can be used as comparative data for future studies evaluating the effectiveness of HAE reduction strategies and injury prevention initiatives. Results indicate that most contact events in rugby union caused either no recorded HAE, or lower-magnitude HAEs, while higher-magnitude HAEs were comparatively rare. On average, HAEs above 40 g occurred approximately once in every 30 tackles, 28 carries and 59 rucks in men, and every 111 tackles, 91 carries and 333 rucks in women. For men's forwards and backs, these HAEs occurred every 60 and 100 min, respectively, and every 200 and 300 min for women's forwards and backs. Incidence was significantly higher in men than women as a result of greater propensity of all contact events to cause HAEs in men, and significantly higher in forwards than backs as a result of greater involvements of forwards in contact. The initial collision stage of the tackle was identified as the highest risk area for HAE mitigation strategies. These findings benchmark HAE exposures in elite-level rugby union for the first time and provide a basis from which to develop strategies aimed at reducing HAEs.

## Policy Implications

The tackle event should remain a focus of strategies aimed at reducing HAE propensity with consideration for both the tackler and the ball carrier. The initial collision of the tackle event should be targeted specifically. Rucks are also a concern due to the high number of events per match. Incidence reported in this study may have implications for position- and sex-specific guidelines on contact load management, due to differences in the incidence of HAEs.

### Supplementary Information

Below is the link to the electronic supplementary material.

Supplementary file1 (PDF 641 kb)

## Declarations

### Data Sharing

All data relevant to the study are included in the article or uploaded as supplementary information. Anonymised data should be available upon reasonable request.
