# Correction to: Instrumented Mouthguards in Elite-Level Men’s and Women’s Rugby Union: The Incidence and Propensity of Head Acceleration Events in Matches

**DOI:** 10.1007/s40279-023-01968-0

**Published:** 2023-11-08

**Authors:** James Tooby, James Woodward, Ross Tucker, Ben Jones, Éanna Falvey, Danielle Salmon, Melanie Dawn Bussey, Lindsay Starling, Gregory Tierney

**Affiliations:** 1 Carnegie Applied Rugby Research (CARR) Centre, Carnegie School of Sport, Leeds Beckett University, Leeds, UK; 2 Sport and Exercise Sciences Research Institute, Ulster University, Belfast, UK; 3 Department of Sport Science, Institute of Sport and Exercise Medicine, University of Stellenbosch, Stellenbosch, South Africa; 4 World Rugby, 8-10 Pembroke St., Dublin, Ireland; 5 Division of Physiological Sciences and Health Through Physical Activity, Department of Human Biology, Faculty of Health Sciences, Lifestyle and Sport Research Centre, University of Cape Town, Cape Town, South Africa; 6 England Performance Unit, Rugby Football League, Manchester, UK; 7 Premiership Rugby, London, UK; 8 Faculty of Health Sciences, School of Behavioural and Health Sciences, Australian Catholic University, Brisbane, QLD Australia; 9 School of Medicine & Health, University College Cork, Cork, Ireland; 10 New Zealand Rugby, Auckland, New Zealand; 11 School of Physical Education Sport and Exercise Sciences, University of Otago, Dunedin, New Zealand

**Correction to: Sports Medicine** 10.1007/s40279-023-01953-7

At the time of online publication, data were inadvertently missing from Fig. 6.

**Fig. 6:**
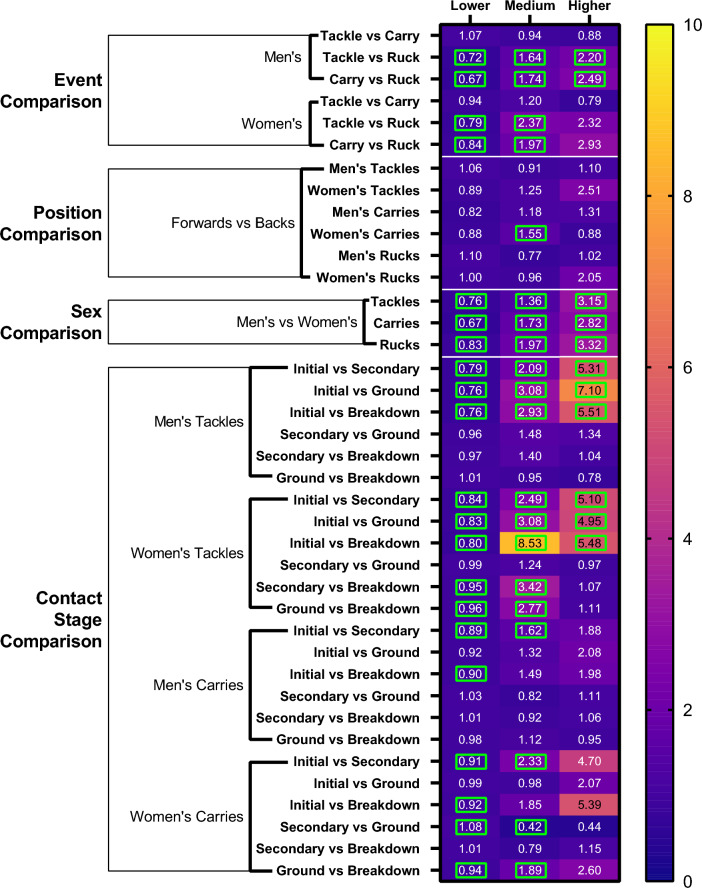
Propensity ratios of tackles, carries and rucks to result in a maximum-magnitude HAE within lower-, medium- and higher-magnitude bands. If no HAE was recorded during a contact event then the maximum HAE was considered to be within the lower band. Comparisons between events, positions and sexes, and contact stages are included. Significant comparisons are indicated by *green boxes*. *HAEs* head acceleration events

The correct Fig. 6 has been copied below

The original article has been corrected.

