# Supplementary material for: Instrumented Mouthguards in Elite-Level Men’s and Women’s Rugby Union: The Incidence and Propensity of Head Acceleration Events in Matches
Source: Sports Med. 2023 Oct 31;54(5):1327–38. doi: 10.1007/s40279-023-01953-7 (PMC11127838; doi:10.1007/s40279-023-01953-7)
Supplement: Supplementary file 1 — Supplementary file1 (PDF 641 kb) [file 40279_2023_1953_MOESM1_ESM.pdf]

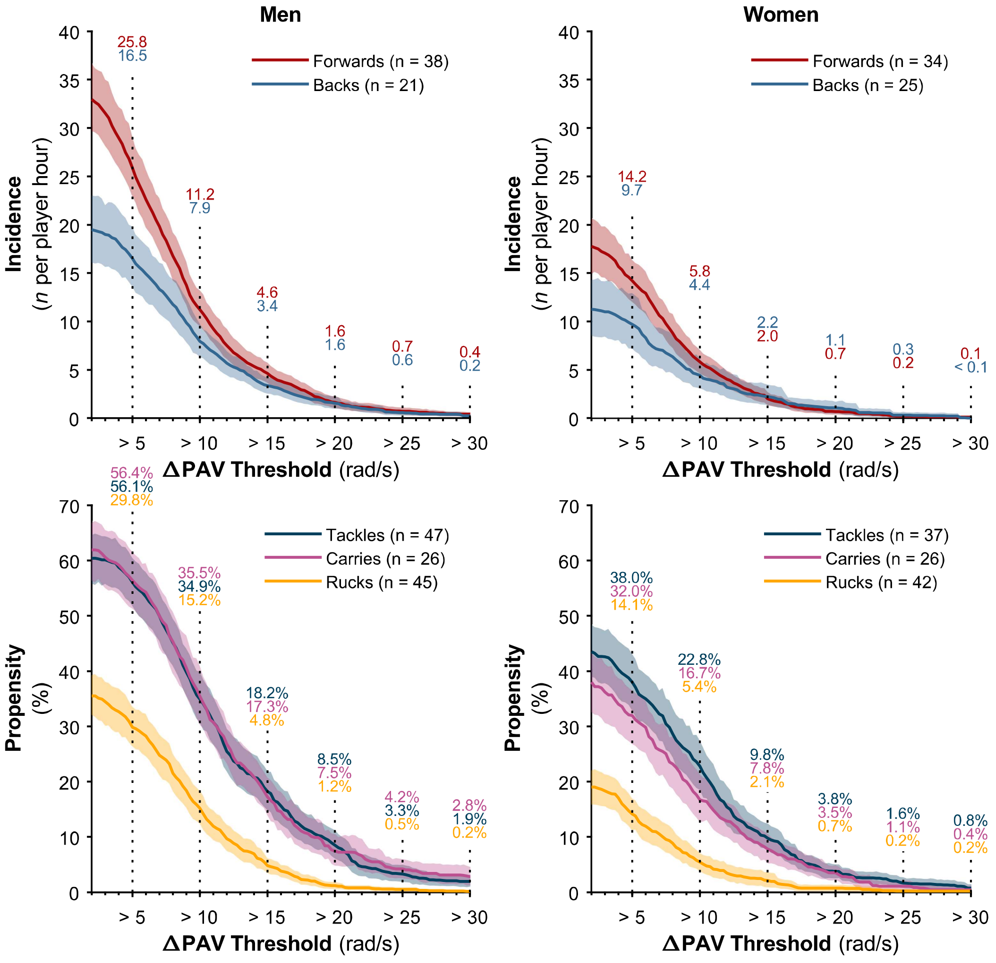

Supplementary Figure 1. The incidence of HAEs and the propensity of tackles, carries and rucks to result in at least one HAE exceeding a given threshold presented across ΔPAV thresholds (5 to 30 rad/s) for men's and women's players. Shaded regions indicate 95% CI. The number of players available to calculate each curve is shown as *n*. Text labels are added at intervals along the curve. ΔPAV was calculated by zeroing x, y, and z components of angular velocity to the trigger point and the peak value was taken from a recalculated resultant curve from the zeroed components.

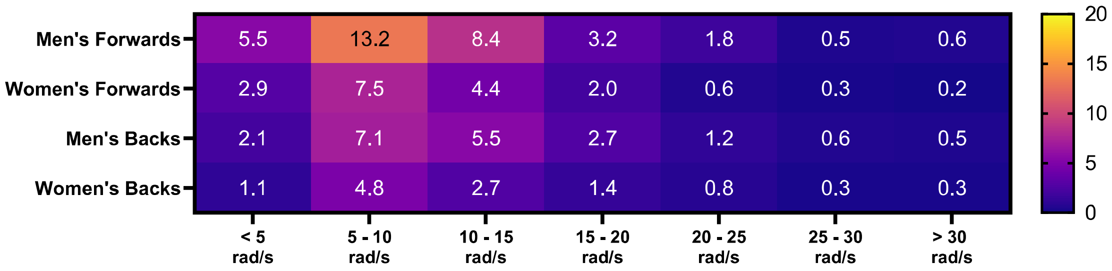

Supplementary Figure 2. Incidence values between ΔPAV thresholds.

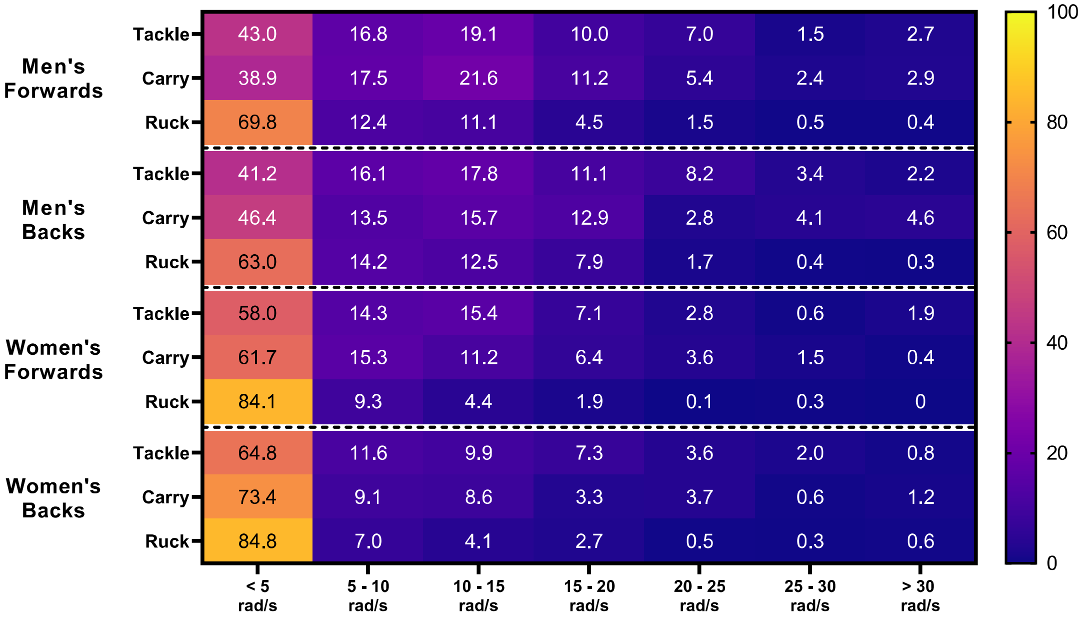

Supplementary Figure 3. Propensity values between ΔPAV thresholds.

	Median (IQR)
**Men's Forwards**	20.97 (8.25 – 28.33)
**Men's Backs**	12.87 (9.00 – 16.69)
**Women's Forwards**	10.59 (8.57 – 15.00)
**Women's Backs**	6.08 (3.56 – 9.90)

Supplementary Table 1. Incidence values presented as median (25th to 75th percentile) as per King et al., 2016[21].
